# Police referrals for domestic abuse before and during the first COVID-19 lockdown: An analysis of routine data from one specialist service in South Wales

**DOI:** 10.1093/pubmed/fdab343

**Published:** 2021-09-25

**Authors:** Graham Moore, Kelly Buckley, Emma Howarth, Anne-Marie Burn, Lauren Copeland, Rhiannon Evans, Lisa Ware

**Affiliations:** Centre for Development, Evaluation, Complexity and Implementation in Public Health Improvement, School of Social Sciences, Cardiff University, Cardiff CF10 3BD, UK; Wolfson Centre for Young People’s Mental Health, Cardiff University, Cardiff CF24 4HD, UK; Centre for Development, Evaluation, Complexity and Implementation in Public Health Improvement, School of Social Sciences, Cardiff University, Cardiff CF10 3BD, UK; School of Psychology, University of East London, London E16 2RD, UK; Department of Psychiatry, University of Cambridge, Cambridge CB2 0SZ, UK; Centre for Development, Evaluation, Complexity and Implementation in Public Health Improvement, School of Social Sciences, Cardiff University, Cardiff CF10 3BD, UK; Centre for Development, Evaluation, Complexity and Implementation in Public Health Improvement, School of Social Sciences, Cardiff University, Cardiff CF10 3BD, UK; Cardiff Women’s Aid, Cardiff CF24 0EJ, UK; RISE Cardiff, Cardiff CF24 0JT, UK

**Keywords:** child/adolescent to parent violence, children, COVID-19, domestic abuse, police

## Abstract

**Background:**

COVID-19 lockdown measures may have led to more, and increasingly severe, domestic abuse. This study examines police referrals to a specialist domestic abuse service in Wales, UK before and during the first lockdown.

**Methods:**

Routine data relating to 2292 police referrals for female adult victim-survivors from December 2019 until July 2020 were analysed and presented in the form of descriptive statistics to monitor changes in referral rates and the profile of those referrals.

**Results:**

There was little increase in the overall volume of police referrals during lockdown, but the proportion assessed as high risk increased, and children became the primary source of third-party referrals, with a higher proportion of reports made by other third parties as restrictions eased. Police reports for cases of Child/Adolescent to Parent Violence (C/APV) occurred almost exclusively during lockdown.

**Conclusions:**

The increase in risk level despite less clear increase in volume may suggest unmet need, with victims less likely to seek help during lockdown other than for more severe instances. Increased reports by children suggest increased exposure of children to domestic abuse during school closure. Unmet need for women and children may have been made visible to services, and acquaintances, as measures began to ease.

## Introduction

The COVID-19 pandemic led to prolonged social distancing restrictions.^1^ In UK nations, from March until June 2020, legislation required people to not leave home without a ‘reasonable excuse’ (e.g. essential work or care for a vulnerable person^2^). Schools closed other than to children of keyworkers and those considered vulnerable.^3^^,^^4^ Accompanied by the slogan of ‘Stay Home, Protect the NHS, Save Lives’,^2^ this aimed to limit social interactions and control infection. While widely supported by the public,^5^ concerns were expressed regarding potential social harms.

Concerns included the impacts on domestic abuse and reduced access to support. The UK Government defines domestic abuse as ‘any incident or pattern of incidents of controlling, coercive or threatening behaviour, violence or abuse between those aged 16 or over who are or have been intimate partners or family members regardless of gender or sexuality. This can encompass but is not limited to psychological, physical, sexual, financial and/or emotional abuse’.^6^ The Department of Health^7^ has recognized that domestic abuse is harmful to adults and children, and a wealth of evidence documents physical and psychological health harms of domestic abuse.^8^^,^^9^ At the forefront of concerns for many were that lockdown would force victim-survivors to ‘Stay Home’ with an abusive partner.^10^^,^^11^ Early in the pandemic, government communications were inconsistent about whether children in shared custody arrangements could move between homes, leading to reported conflict between separated parents.^12^ Provisions were made in law recognizing that seeking refuge was ‘a reasonable excuse’ to leave home.^13^ The UK and Welsh Governments backed two campaigns (You Are Not Alone^14^ and Home Shouldn't Be a Place of Fear^15^) to enable victim-survivors to find support, while traditional pathways and services were unavailable or unsafe. Internationally, evidence indicates that concerns regarding growth in domestic abuse were well founded.^16^^,^^17^

One study from Wales reports no evidence of increased volume of hospital presentations for violence in the home during the first lockdown.^18^ However, it is likely that many victims avoided hospital during the pandemic or that much increased abuse did not result in hospitalization.^19^ Examining change over time in police referrals offers another means to examine trends in incidence and nature of domestic abuse before and during the pandemic. It also offers insights into impacts on exposed third parties, including children, by allowing exploration of whether numbers of children actively making police contact for episodes of domestic abuse increased during the pandemic compared with before and after lockdown periods. The pandemic and associated lockdown may have had significant implications for the nature of abuse,^20^ and implications for family members, such as children, if exposed to abuse while spending more time at home and cut off from avenues of support during periods of school closure. Increases in family conflict may have given rise to other forms of domestic abuse, including violence by adolescent children toward their parents (‘Child and Adolescent to Parent Violence’ or C/APV), which commonly go unreported outside of lockdowns.^21^

During the pandemic, help-seeking and pathways of referral to services may have changed. In the UK, a multi-agency response is considered best practice, routinized through the Multi-Agency Risk Assessment Conference process and established through Home Office policy and legislative requirements that seek to embed partnership working and information sharing to tackle domestic abuse.^22^ Arguably the multi-agency response to domestic abuse is more important than ever in a pandemic when victim-survivors’ contact with some agencies will be limited. Understanding experiences and pathways of those seeking support during the pandemic is vital in order to meet the needs of victim-survivors and their families. Concerns have been raised about the impact the growth of domestic abuse would have on third sector services, with 88% of services reporting an increase in demand, and 41% fearing they were unable to meet this increase in demand.^23^ From a recent survey of frontline professionals, it was suggested that the increase in referrals to services was not necessarily an increase in victims, but increased severity and shortage of available coping mechanisms.^23^

This paper reports exploratory analysis of routine data from a domestic abuse organization in Cardiff, Wales, examining trends in police referrals of adult women for domestic abuse in the months prior to and during the first lockdown. We address the following research questions, which arose through discussions with practice partners in an ongoing study focused on supporting children’s recovery from domestic abuse:

(i) Did the volume, and/or risk level, of police referrals change over time through the first lockdown period relative to baseline trends?
(ii) Did the source of police reports (i.e. by the victim or third parties) change through the first lockdown period?
(iii) Who were the primary perpetrators of reported abuse episodes against adult women during lockdown?

## Methods

### Study design and measures

Data on police referrals of adult (i.e. ≥18 years or over) women to specialist domestic abuse service RISE-Cardiff were recorded before and during the first lockdown. Referrals were risk-assessed by police using the Safelives DASH Risk Checklist,^24^ an evidence-based risk assessment tool widely used to help frontline practitioners identify high risk cases of domestic abuse, stalking and ‘honour-based’ violence. Monthly data were provided to the research team in an anonymized form for December 2019 to July 2020. The dataset provided 2292 referrals across an 8-month period comprising 3 months prior to lockdown (December 2019 to February 2020), lockdown itself (March 2020–June 2020) and the first month after easing began (July 2020). March and June represent transition periods into and out of lockdown. Data were recorded in open fields on an Excel spreadsheet, and retrospectively coded for analysis, with meetings between the Cardiff University and RISE-Cardiff teams held to sense check interpretations of the coding. Fields derived from the data were: (i) month of referral; (ii) source of police report (coded as victim, family, friend, professional, neighbour, other third party, perpetrator or child); (iii) relationship of person committing the abuse, coded as partner, ex-partner or child to parent (i.e. cases where the person reported as having committed abuse was <18 years old) or other. Ethical approval was provided by the Cardiff University School of Social Sciences Research Ethics Committee.

**Fig. 1:**
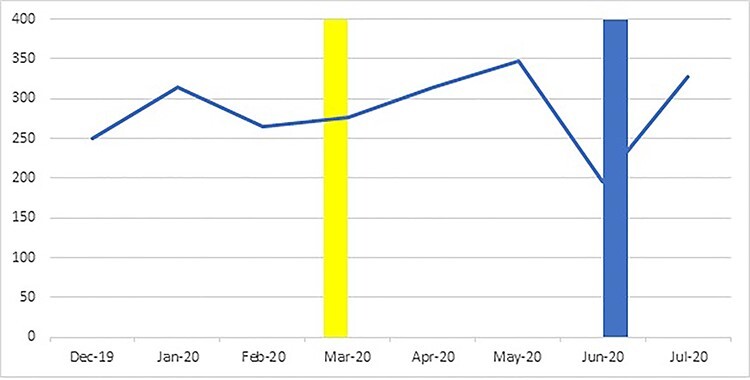
Total number of referrals by month, before during and after ‘lockdown’ (yellow bar = beginning of lockdown; blue bar = beginning of easing of lockdown).

### Analysis

Our analysis focuses on change over time in volume and nature of referrals prior to and during COVID-19 lockdown restrictions. Given the small number of time points and the changing nature of pandemic control interventions over the study time period, with monthly data available for 3 months prior to the introduction of the harshest ‘lockdown’ measures, and the 2 months during these, it was not possible to undertake a formal interrupted time series analysis. Hence, analyses take the form of descriptive statistics, charting change over time in the lead up to, during and immediately after lockdown. We graphically present the number of referrals per month. We then report the total number of referrals received from each source overall and present percentages for each category by month. Finally, we present percentages of cases overall and per month relating to the relationship between perpetrator and victim, charting percentages over time as well as percentages of all referrals per month classified as ‘high risk’ referrals. We used chi-squared tests to assess the significance of differences in risk level, reporting party and perpetrator across 3 periods—pre-lockdown (December to February), lockdown (April and May) and post lockdown (July)—excluding months which involved transition in and out of lockdown.

## Results

### Total number of referrals by month

Overall, 2292 referrals were recorded between December 2019 and July 2020 (mean = 286.5 per month). The highest number was reported in May, with the lowest in June and little clear evidence of a time trend (Fig. 1).

### Risk level

**Fig. 2:**
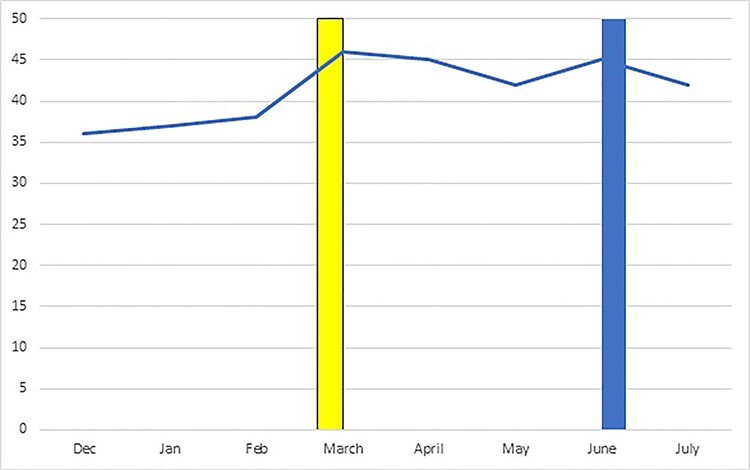
Percentage of referrals classed as ‘high risk’ according to police risk assessment, by month (yellow bar = beginning of lockdown; blue bar = beginning of easing of lockdown).

Overall, 41.3% (*N* = 946) of referrals were classed as ‘high risk’. The percentage of referrals assessed as high risk increased from slightly more than 1 in 3 in the months prior to lockdown, to almost half in the month of lockdown, remaining above the baseline level through lockdown (Fig. 2). Chi-squared analysis indicated significant differences between time periods in the percentage of referrals classed as high risk (Chi-squared = 6.9; *P* = 0.032).

### Reporting party

Most reported cases involved police contact initiated by victim-survivors (*N* = 1951; 85.2%). The next most common category was unidentified third parties (*N* = 133; 5.8%). Identified third party reporters included neighbours (*N* = 74; 3.2%), professionals (*N* = 59; 2.6%), children (*N* = 32; 1.4%), friends (*N* = 21; 0.9%), other family members (*N* = 17; 0.7%) and in rare cases, the perpetrator themselves (*N* < 5).

**Fig. 3:**
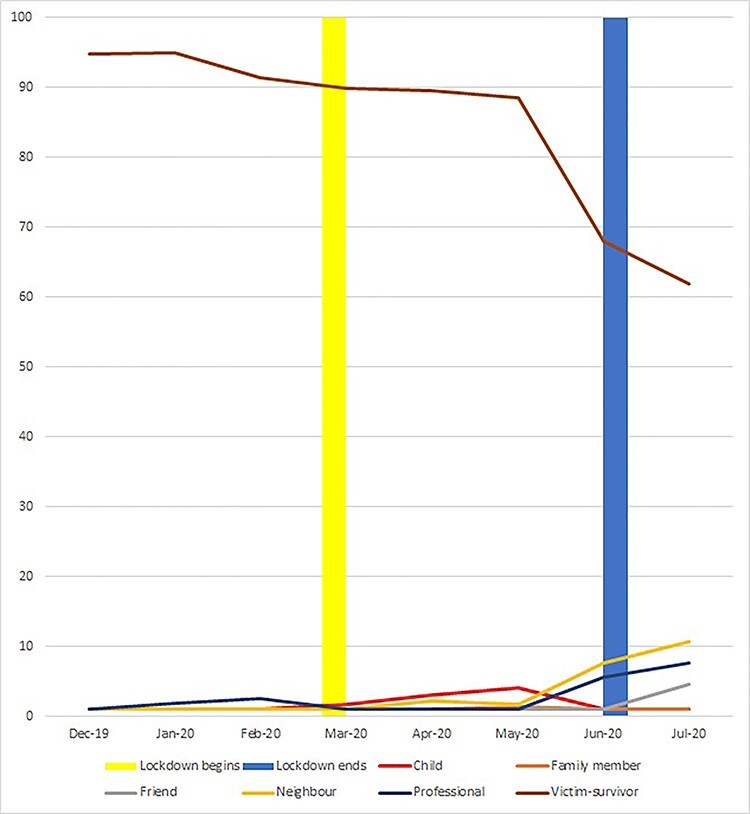
Percentage of self and third party police contacts by month and category of reporter (due to very small numbers in some categories, where *n* < 5, value is set to 1%; yellow bar = beginning of lockdown; blue bar = beginning of easing of lockdown).

While in all months, most police reports were made by the victim-survivor, this declined over time, falling from 94.8 to 91.3% in the months prior to lockdown, and more markedly as lockdown measures began to ease, to 68.0% in June and 61.9% in July (Fig. 3). Two separate salient patterns emerged in relation to third party referrals through lockdown and beyond. Reports by children occurred almost exclusively during lockdown months of April and May or months of transition into and out of full lockdown. Indeed, children became the primary source of third party reports during the lockdown months. In contrast, reports by friends, neighbours and professionals remained low during lockdown but grew on the easing of restrictions in June, with reports by children reducing again. Chi-squared analyses indicated that significant differences between time periods in by whom abuse was reported (Chi-squared = 329.7; *P* < 0.001).

### Relationship to victim-survivor of the person committing the reported abuse

**Fig. 4:**
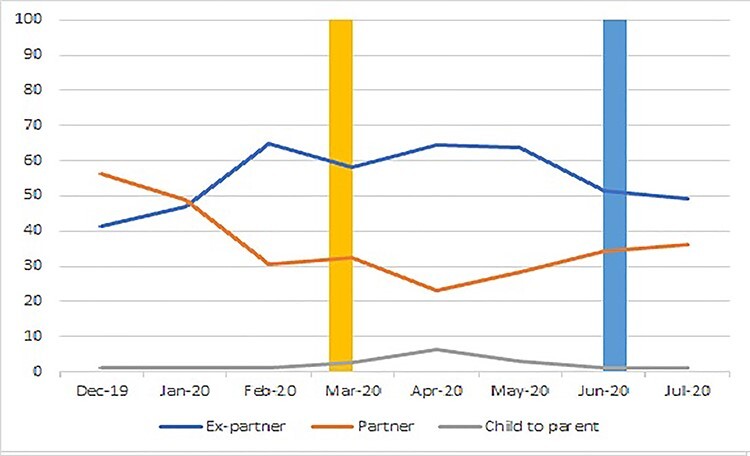
Percentage of cases in which the person committing the reported abuse was a partner, ex-partner or a child, by month (due to very small numbers in some categories, where *n* < 5, value is set to 1%; yellow bar = beginning of lockdown; blue bar = beginning of easing of lockdown).

Across the time period as a whole, in a slight majority of cases, the reported perpetrator was an ex-partner (55.5%; *n* = 1272), with most remaining cases relating to a current partner (36.0%; *n* = 826). In the first 2 months (pre-lockdown), abuse by current partner was more common, although throughout lockdown, this was reversed, with more than 60% of cases relating to an ex-partner in the full lockdown months of April and May (Fig. 4). However, this crossover occurred before lockdown. The next most common relationship type recorded related to reports of child to parent abuse, of which 40 (1.8% of all cases) cases were reported during the study period; most occurred in ‘full’ lockdown months of April and May (75.0%; *N* = 30). Chi-squared analysis indicated significant variation between time periods in the relationship to the victim-survivor of the person committing abuse (chi-squared = 153.4; *P* < 0.001).

## Discussion

### Main findings of this study

Our analysis contributes to the complex picture facing domestic abuse services during the COVID-19 pandemic.^25^ Police referral numbers did not increase substantially, but a larger proportion of referrals were assessed as ‘high risk’ during lockdown. There were important changes in the nature of police referrals, including the person reporting the abuse to the police and their relationship to the victim-survivor. We found limited professional contacts with police until after lockdown, along with increases in contacts made by children. During lockdown, children became the primary source of third party referrals. As lockdown eased, other third parties, including professionals, friends and neighbours, took up a greater role in reporting abuse. Our data provide some evidence of increased C/APV, accounting for 2% of police contacts in total, but occurring mostly within lockdown months.

### What is already known on this topic

Domestic abuse has been one of the key social harms raised as a concern during the pandemic and associated restrictions globally.^11^ Data on prevalence are complicated by reliance on individual service use data as proxies for domestic abuse, different services likely revealing different aspects of this complex picture. Hospital data indicate no increases in hospital presentations for violence in the home during the first lockdown in Wales.^18^ A recent survey of professionals^23^ showed that 9 out of 10 services reported increased demand, yet similar data from June 2020^26^ suggest only 38% of services reported an increase in demand in the first lockdown period. Despite concerns of increasing prevalence during the pandemic and reports of increased calls to the National Domestic Abuse Helpline,^27^ women’s self-reports decreased during this time, which may represent the lack of opportunity to contact support services for women confined with abusers.^20^ Hence, while all service use data are likely to underestimate changes in prevalence, these provide means of understanding changes in the nature of abuse. Concerns have been raised about exposure of children and young people to domestic abuse within their households as a result of spending more time at home, while rising family conflict may also have given rise to increases in C/APV; in June 2020, 14% of frontline domestic abuse services reported an increase in C/APV abuse.^26^ Another study found that 69% of practitioners reported that they had seen an increase in referrals for families experiencing C/APV.^21^ This has been raised in the media as a key concern,^28^ in addition to other forms of domestic abuse, such as elder abuse and revenge porn.^29^

### What this study adds

This study adds to limited evidence on help-seeking of families experiencing domestic abuse in the UK during the pandemic. Our analysis shows little increase in the volume of referrals, although referrals tended to be higher risk during lockdown. This perhaps indicates a tendency for women and third parties to be less likely to make police contact for domestic abuse during lockdown except for severe instances, pointing to unmet need and underestimation of growth in prevalence. As referrals into the service were from police, this adds complexity to the accepted picture of increased occurrence of domestic abuse, highlighting that some mechanisms of help-seeking (such as contacting the police) may have been less used during lockdown periods, other than for more severe cases.

This study adds to concerns about increased exposure of children to domestic violence.^25^ In ‘normal’ times, schools represent a place of safety as well as an avenue to disclose difficulties at home. However, during lockdown, this source of support was unavailable for most children.^30^ Coupled with a lack of contact with other helping professionals, this may have resulted in more children reporting to police. These results may also reflect an increase in the severity or frequency of abuse during the pandemic, which is known to be associated with increased adult rates of contacts with police.^31^ That children increasingly initiated contact may reflect constraints placed on survivors by the pandemic.^32^ These results highlight the importance of prioritizing support of children and young people impacted by domestic abuse in the pandemic recovery who were not reached by specialist services,^23^ but were, as our analysis shows, the main source of third party referrals to police during the lockdown months. Specialist services for children experiencing domestic abuse need to be prioritized for funding, together with training for other agencies, such as the police, to help them understand the impact of domestic abuse on children, increase their comfort in talking to children about abuse and importantly how to refer them to specialist support.

Our finding relating to the rise of third parties making police reports as the lockdown period ended also supports the calls made by specialist domestic abuse charities^23^ to extend campaigns such as ‘Ask for ANI’ to other services such as banks, supermarkets and to encourage friends, family and neighbours to ‘Reach In’ to those who may be experiencing abuse.^32^ However, frontline domestic abuse services have expressed concern over partner agency capacity,^23^ particularly in relation to the delay in accessing mental health services for both adults and children. Our findings have highlighted the importance of mental health services for Children and Young People being adequately funded to meet this increased demand, in order for specialist domestic abuse services to refer for counselling and other support that can be accessed in a timely way and support families’ recovery from abuse. The multi-agency response to domestic abuse means partner agencies that work alongside specialist domestic abuse services need to have increased awareness and capacity to support in relation to the issues our findings raised, such as children witnessing domestic abuse and C/APV.

Finally, this study adds to a limited evidence base on the increase in reporting of C/APV in the pandemic. An increase in parent–child interaction due to school and workplace closures may have instigated abuse, or abuse may have escalated in severity and/or frequency during lockdown. Our finding supports calls made by other research^21^ for increased mental health support for parents and children who may have found the lockdown traumatic, and targeted interventions to improve familial relationships, particularly parent–child, that may have been harmed by lockdown.

### Limitations of this study

Data are from a single service in an urban district in South Wales and hence should be treated as exploratory. The observational design means changes cannot be causally attributed to lockdown measures. Data pertain specifically to police, rather than all referrals: given that most DVA victims do not report (directly or indirectly) to police,^33^ these provide only a partial picture of need and demand for services during this time.

## Conclusion

Our data indicate changes in severity and nature of referrals received by one South Wales charity during this time. Prior to lockdown, almost all police reports came from women experiencing abuse. During lockdown, children became the primary source of third-party referrals, perhaps indicating a higher degree of exposure due to school closures, and a lack of opportunities to disclose to other professionals. In absolute terms, the number of reports by children was small given our exploratory focus on one service in Wales. However, if this pattern is replicated in other parts of Wales and the UK, this likely constitutes a substantial unmet need among children exposed to domestic abuse during lockdown. As lockdown measures began to ease, other actors, such as neighbours, friends and professionals substantially increased their share as reporters of domestic abuse. This perhaps points to a significant degree of unmet need for women and children during lockdown, made visible to services, and acquaintances, as measures began to ease. Given that there have been two further lockdown periods since these data were obtained, there is an urgent need to better understand impacts of prolonged restrictions for women and children, to understand how to support help-seeking behaviour and recovery.

## Funding

This study was undertaken by a team working on a related study, Family Recovery after Domestic Abuse (FReDA), which is funded by the National Institute for Health Research via the Public Health Research funding committee (NIHR127793). The views expressed in this publication are those of the authors and do not necessarily reflect those of the UK NHS, the National Institute for Health Research or the Department of Health for England. The project was undertaken with the support of The Centre for the Development and Evaluation of Complex Interventions for Public Health Improvement (DECIPHer), a UKCRC Public Health Research Centre of Excellence. Joint funding (MR/KO232331/1) from the British Heart Foundation, Cancer Research UK, Economic and Social Research Council, Medical Research Council, the Welsh Government and the Wellcome Trust, under the auspices of the UK Clinical Research Collaboration, is gratefully acknowledged. This project was also supported by its successor The Centre for Development, Evaluation, Complexity and Implementation in Public Health Improvement (DECIPHer) at Cardiff University, funded by Welsh Government through Health and Care Research Wales.

## Notes

**Graham Moore,** Professor of Social Sciences & Public Health

**Kelly Buckley,** Research Associate

**Emma Howarth,** Senior Lecturer

**Anne-Marie Burn,** Research Associate

**Lauren Copeland,** Research Associate

**Rhiannon Evans,** Senior Lecturer in Social Sciences and Health

**Lisa Ware,** Support Services Manager
